# 2025: status of cardiac xenotransplantation including preclinical models

**DOI:** 10.3389/frtra.2025.1568910

**Published:** 2025-04-15

**Authors:** Guerard W. Byrne, Christopher G. A. McGregor

**Affiliations:** ^1^ Department of Surgery, Experimental Surgical Services, University of Minnesota, Minneapolis, MN, United States; ^2^ Institute of Cardiovascular Sciences, University College London, London, United Kingdom

**Keywords:** xenotransplantation, cardiac, antibody-mediated rejection (ABMR), decedent model, genetic engineering

## Abstract

Xenotransplantation offers an opportunity to radically change the availability of organs for life-saving human transplantation. Great progress has been made in porcine donor genetic engineering to reduce the immunogenicity of pig organs and potentially enhance their resistance to antibody-mediated rejection. There is also growing insight into more effective immune suppression regimens. These advances have improved the duration of cardiac xenograft survival in non-human primates over the last decade and supported the recent approval of the first-in-human clinical use of pig hearts and kidneys for transplantation. This review critically examines preclinical and clinical results in cardiac xenotransplantation. We identify challenges that remain to achieve consistent and durable clinical graft survival. We discuss the relative value of preclinical non-human primate and human decedent transplant models to optimize patient cross-matching, immune suppression, postoperative monitoring, and graft survival.

## Clinical xenotransplantation

The concept of xenotransplantation (Xtx) is centuries old, but the modern era can be considered to have begun with the work of Reemtsma et al. and Starzl et al. who each reported a series of non-human primate (NHP) kidney to human Xtx in the 1960s (1, 2). The first human cardiac xenotransplantation (CXtx) was performed by Hardy et al. in 1964 transplanting a chimpanzee heart in a critically ill patient who survived only hours because the donor heart was too small relative to the recipient (3). In 1977, Barnard et al. reported heterotopically placed NHP hearts in two patients with postcardiotomy heart failure. Survival was 5 h in 1 and 4 days in the other, but both donor hearts were too small to support the circulation effectively (4). A notable event was the NHP to human neonate CXtx by Bailey et al. at Loma Linda in 1983. Survival was for 20 days with excellent early function, but the organ failed due to a terminal diagnosis of antibody-mediated rejection (AMR) from a donor-recipient blood group mismatch (5).

Although NHPs are attractive donor candidates because of their close immunological and physiological homology to humans, these early clinical cases highlight their limitations as an acceptable long-term solution to the organ shortage. The largest NHPs required to match adult human cardiac size are on the endangered species list, and their use creates additional ethical and societal concerns. NHPs also represent a high risk of potential zoonotic infectious disease, and, largely for this reason, their use for Xtx was effectively banned in 1999 (6).

Over the last two decades, although phylogenetically separated from humans by ∼70 million years, the pig has emerged as the potential species of choice as a source for human organs (Table 1). Compared to NHPs, the broad availability of pigs, their reproductive performance, and the ability to genetically engineer this species are advantages that outweighed the pig's increased immunological and physiological dissimilarities in anatomy, physiology, and growth. Using an array of genetically engineered (GE) donor pigs and progressively evolving immune suppression strategies, experimental preclinical CXtx in NHP recipients (initially heterotopic and subsequently orthotopic) has shown great improvements in efficacy (Tables 2, 3). Based on these results, human Xtx studies have recently been approved under FDA single-patient expanded access protocols (EAPs) using GE donor pigs to treat severely ill patients with a high risk of mortality and no other alternatives. In 2022 and 2023, one patient each year with terminal heart failure at the University of Maryland (UMD) underwent the world's first clinical CXtxs using GE pig donors (7, 8). Both patients developed abrupt onset diastolic heart failure, on Days 47 and 35, respectively, leading to their deaths after 60 and 40 days, principally from AMR, sooner than might have been expected from the UMD's excellent results in NHPs (9–11). These initially successful but short-lived clinical procedures at UMD provided important data but were necessarily focused on patient survival rather than prospective, protocolized research studies. Of relevance, the first, living human kidney Xtx from a GE pig was done in March 2024 in Boston (12). After a good initial recovery, the patient died suddenly after 47 days, again sooner than expected, with timing in keeping with the death of the two UMD CXtx patients at 60 and 40 days.

**Table 1:** Porcine donors for cardiac xenotransplantation.

Advantages	Disadvantages
Organ size can match humans	Phylogenetic separation from humans
Established commercial husbandry techniques	Discordant transplant species with high antigenicity
Short gestation (4 months) rapid herd expansion	Hyperacute rejection
Low age of sexual maturity (6 months)	Accelerated growth rate
Large litters (up to 15 piglets)	Biochemical incompatibilities (coagulation, homeostasis, cytokines, chemokines, hormone, and hormone receptors)
Well-established genetic engineering and cloning methods	
Can be maintained in a specific pathogen-free environment	
Zoonoses appear less likely than NHP donors	
Societally more acceptable than NHP donors	

**Table 2:** Preclinical heterotopic cardiac xenotransplantation.

	Heterotopic NHP cardiac xenotransplantation			
	Earlier immune suppression		Co-stimulation blockade	
Donor	CsA/CyP/steroid^a^	ATG/CD20/tacrolimus/sirolimus^a^	ATG/anti-CD154, CD-20/MMF^a^	ATG/anti-CD40, CD20/MMF^a^
WT grafts with:	0.05 (0.04d; *n* = 3) ref ( 18 )			
	0.06d (na) ref ( 19 )			
(A) CVF	2.8d (na)^A^ ref ( 19 )			
(B) Plasmapheresis	17.5d (3.8d; *n* = 5)^A,B^ ref ( 20 )			
(C) sCR1	3.75d (3.0d; *n* = 5)^C^ ref ( 21 )			
(D) Immunoapheresis	32d (21d; *n* = 3)^C^ ref ( 22 )			
or Gal-specific polymer	25d (8d; *n* = 7)^A^ ref ( 23 )			
	15d (13.5d; *n* = 2)^D^ ref (24)			
WT hCRP grafts	5.4d (3.5d; *n* = 4) ref ( 18 )	53 (20.5d; *n* = 18)^B^ ref ( 33 )	139d (27d; *n* = 8)^B^ ref ( 37 )	
Alone or with:	2.8d (1.5d; *n* = 2) ref ( 25 )	109 (19d; *n* = 30)^B^ ref ( 34 )	11 (7d; *n* = 5)^B^ ref ( 38 )	
(A) Immunoapheresis	23d (5d; *n* = 9) ref ( 26 )	113 (76d; *n* = 9)^B^ ref ( 35 )		
(B) Gal-specific polymers	11d (7d; *n* = 3) ref ( 27 )	137 (96d; *n* = 7)^B^ ref ( 36 )		
(C) Gal-specific	29d (7.5d; *n* = 6)^A^ ref ( 28 )			
Immune absorption	36d (28.5d; *n* = 10)^B^ ref ( 29 )			
	39d (33d; *n* = 4)^C^ ref ( 30 )			
	34d (9d; n-5) ref ( 32 )			
	62d (40d; *n* = 10) ref ( 32 )			
GTKO grafts		128d (21.5d; *n* = 5) ref ( 45 )	179d (78d; *n* = 8) ref ( 44 )	
			56d (23.5d; *n* = 4) ref ( 46 )	
			14d (14d; *n* = 3) ref ( 47 )	
GTKO;hCRP grafts		52d (28d; *n* = 5) ref ( 45 )	33d (20.5d; *n* = 4) ref ( 48 )	98d (70d; *n* = 3) ref ( 49 )
			236d (71d; *n* = 9) ref ( 11 )	28d (21d; *n* = 3) ref ( 10 )
				149d (83d; *n* = 6) ref ( 10 )
GTKO;hCRP;TBM Grafts			130d (99d; *n* = 3) ref ( 46 )	945d (298d; *n* = 5) ref ( 9 )
			5d (3d; *n* = 3) ref ( 79 )	
Multigene donor grafts			393d (13d; *n* = 3) ref ( 79 )	
			243d (176d; *n* = 2) ref ( 79 )	

^a^ Table 2 shows the longest organ survival and [median survival and group size (*n*)]. Immune suppression lists the dominant components. See footnotes for Table 3 for more details.

**Table 3:** Preclinical orthotopic cardiac xenotransplantation.

	Orthotopic NHP cardiac xenotransplantation			
	Organ preservation and immune suppression			
	Standard static perfusion			Non-ischemic continuous perfusion
Donor	CsA/CyP/steroid ^a^	ATG/CD20/tacrolimus/sirolimus ^a^	ATG/anti-CD20/anti-CD40 a/o CD40l/MMF ^a^	ATG/anti-CD20/anti-CD40 a/o CD40l/MMF ^a^
WT grafts				
With organ perfusion to	16d (5.5d; *n* = 8) ref ( 90 )			
absorb anti-pig antibody	19d (18d; *n* = 3) ref ( 91 )			
WT;hCRP grafts	9d (2.4d; *n* = 10) ref ( 92 )			
alone or	9d (5d; *n* = 5) ref ( 93 )			
(A) Gal-specific polymers	39d (n.a.; *n* = 1) ref ( 94 )			
	20d (12d; *n* = 4) ref ( 95 )	57d (40d; *n* = 3)^A^ ref ( 39 )		
	25d (5d; *n* = 4)^A^ ref (96)			
	25d (1.2d; *n* = 13)^A^ ref ( 97 )			
GTKO;hCRP;TBM Grafts			30d (1d; *n* = 5) ref ( 85 )	40d (22.5d; *n* = 4) ref ( 85 )
			241d (45d; *n* = 4) ref ( 102 )	195d (90d; *n* = 5) ref ( 85 )
				195d (90d; *n* = 8) ref ( 84 )
				57d (16.5d; *n* = 4) ref ( 83 )
Multigene donor grafts			1.6d (0.8d; *n* = 6) ref ( 99 )	8d (3.2d; *n* = 4) ref ( 83 )
				95d (90d; *n* = 2) ref ( 83 )
				264d (223d; *n* = 2) ref ( 83 )

^a^ Tables 2 and 3 show the longest organ survival and [median survival and group size (*n*)]. Immune suppression lists the dominant components. Superscripts A-D in immune suppression columns denote additional treatment variations listed in the corresponding Donor column. Most experiments also included an array of other agents to control, thrombosis, inflammation or infection. CVF, cobra venom factor; sCR1, soluble CR1 receptor; CsA, cyclosporin; CyP, cyclophosphamide; ATG, antithymocyte gammaglobulin; CD20, rituximab; MMF, mycophenolate mofetil; na, not applicable.

This review examines progress in preclinical CXtx and identifies remaining challenges that appear, at present, to limit consistent and durable clinical graft survival. In light of recent research using human decedent recipients in CXtx, we discuss the potential relative contributions that further preclinical research in NHPs and in the unique human decedent model can make to optimize patient cross-matching, immune suppression, postoperative monitoring, and graft survival.

## Preclinical cardiac xenotransplantation in NHPs: heterotopic transplantation

### The role of antibody and complement

CXtx is limited by AMR manifest as complement-dependent vascular endothelial cell (EC) cytotoxicity, antibody-dependent cell cytotoxicity, and chronic EC activation (13, 14). Consequently, ongoing preclinical CXtx research has been focused on overcoming this recalcitrant immunological barrier using initially a heterotopic non-working heart transplant model and subsequently an orthotopic life-supporting model. The porcine alpha-galactosyltransferase gene (GGTA-1) produces high levels of galactose α1,3, galactose (Gal), a terminal carbohydrate modification (15). Humans and Old World NHPs do not produce the Gal antigen and, in response to gut microfloral stimulation, produce high levels of anti-Gal antibody (16). This anti-Gal antibody induces hyperacute rejection of pig organs within 24 h of transplantation in these species (17). For this reason, early human CXtx studies in NHPs using Gal-positive donor hearts focused on preventing hyperacute rejection using combinations of systemic complement inhibition (cobra venom factor, soluble CR1) or antibody absorption and cyclophosphamide-based immune suppression. Survival of these transplants was limited (18–24) (Table 2). These studies helped clarify the essential role of antibody and complement in xenograft rejection (Table 2).

### Early genetically modified donor pigs

The first GE donor pigs were produced to express high levels of human complement regulatory genes (hCRPs) (CD46, CD55, and/or CD59). These Gal-positive transgenic animals were designed to avoid the use of systemic complement inhibition by creating an intrinsic barrier to vascular complement activation and thereby enhancing the donor organ resistance to AMR (18, 25–30). The results demonstrated that high expression of hCRPs was often sufficient to abrogate the need for systemic complement inhibition and largely prevent hyperacute rejection (31), but was not effective at preventing a posttransplant-induced antibody response and ensuing AMR. Only with high levels of cyclophosphamide could the median survival of CD55 transgenic hearts extend to 40 days (32).

### Control of anti-Gal antibody and non-gal-mediated rejection

Immunoabsorbent Gal-specific columns and non-antigenic polymers of Gal antigen were developed to extract or block circulating anti-Gal antibodies and used in conjunction with hCRP transgenic donors under a variety of immune suppressive regimens (29, 30, 33–38) in both preclinical heterotopic and orthotopic transplant models (Tables 2, 3). Gal-specific columns were effective in removing anti-Gal antibodies but did not limit antibody rebound and their use significantly complicated the transplant process. Gal-specific immunopheresis increased graft survival to a median of circa 1 month (30). The use of Gal-polymers with transgenic donors further improved graft survival to a median of 2–3 months (29, 33–38), but this benefit was only achieved with more effective immune suppression (38). Gal-polymers were the first methodology to achieve a >90-day median survival in the heterotopic model and nearly 2-month survival in an early orthotopic transplant (36, 39). The success of these polymers was probably due to their ability to block the posttransplant induction of anti-Gal antibodies (36, 37, 39, 40). These results provided the first evidence that AMR of CXtx, in the absence of an induced anti-Gal antibody response, was now targeted to other (non-Gal) porcine antigens (29, 34).

### Gal-knockout donor pigs and non-Gal antibody-mediated rejection

Definitive proof that antibody to non-Gal porcine antigens was affecting CXtx rejection required the establishment of Gal-free pigs with a targeted mutation in the porcine GGTA-1 gene which encodes the alpha-galactosyltransferase (GTKO) required for synthesis of the Gal-glycan (41–43). Transplantation of GTKO donor hearts eliminated the need for Gal-polymers or anti-Gal antibody absorption and eliminated the posttransplant induction of anti-Gal antibodies. Survival of GTKO hearts was comparable to the best results obtained with Gal-polymers and transgenic donor organs (44–47). When hCRPs were included in the GTKO background, CXtx results remained variable, but maximal cardiac survival substantially increased to 236 days using co-stimulation blockade immune suppression (10, 11, 45, 48, 49). Genetic engineering of the GGTA-1 gene has been successfully done in mice, rabbits, and sheep, and GTKO pig tissues have also been approved for consumption in persons with alpha-Gal sensitivity (50–53). GTKO pig tissue, due to its reduced antigenicity, has also been proposed as an improved source of tissue for replacement heart valves (54, 55).

### Non-Gal antigens

CXtx even with GTKO donor pigs remains subject to AMR manifest as complement-dependent vascular EC cytotoxicity, antibody-dependent cell cytotoxicity, and chronic EC activation (13, 14). Early analysis of non-Gal immune responses in NHPs suggested a pan-pig response to porcine proteins (56). This is consistent with later proteomic studies which identified a range of potential target proteins with some evidence of immunodominant antigens (57–59). Further analysis of preclinical NHP serum samples after CXtx identified specific immunogenic porcine proteins and an unanticipated immunogenic glycan (SDa) encoded by the porcine beta-1,4-N-acetyl-galactosaminyltransferase 2 (B4GALNT2) (60, 61). SDa is in the polyagglutinable human SID blood group. Most individuals have low levels of anti-SDa IgM which agglutinates red blood cells with high SDa levels (62–64). Additionally, human antibodies to N-glycolylneuraminic acid (Neu5Gc)-modified glycans are expected to contribute to clinical CXtx. The anti-Neu5Gc antibody is not present in NHP recipients because they, like pigs, have an active cytidine monophosphate-N-acetylneuraminic acid hydroxylase gene (CMAH) required for Neu5Gc synthesis. Glycans with Neu5Gc modifications are however known to be immunogenic in humans and have long been recognized to contribute to clinical serum sickness (65–67).

The xenogeneic porcine glycans Gal, SDa, and Neu5Gc have all been targeted by genetic engineering to produce pigs with reduced antigenicity. Cells from pigs engineered with mutations to eliminate expression of these glycans show progressively reduced human antibody binding as each glycans is deleted (GTKO, single KO; GTKO/B4GALNT2KO, double knockout; and GTKO/B4GALNT2KO/CMAHKO, triple knockout). Approximately 30% of patient samples show no apparent reactivity to porcine triple knockout cells (68). Unexpectedly in NHPs, which make Neu5Gc-modified glycans and do not make antibodies to Neu5Gc, the Gal and SDa double knockout cells show the least antibody binding while triple knockout cells lacking Gal, SDa, and Neu5Gc show higher antibody binding. This has suggested to some that a fourth cryptic antigen is recognized by NHP antibodies due to the lack of porcine Neu5Gc expression (69). The disparity in antibody reactivity between NHPs and humans to Neu5Gc-modified glycans and to double vs. triple knockout porcine cells complicates advancement to the clinic as the optimal donor pig may be different between NHPs and humans. In any case, the elimination of these three glycans has dramatically reduced human porcine donor immunogenicity but has not prevented preclinical (70, 71) or clinical (7, 8, 72, 73) xenograft AMR. This suggests that additional protein or glycan antigens remain and contribute to xenograft rejection.

Aside from anti-glycan antibodies, it is apparent that human serum is reactive to some porcine proteins. Some but not all highly sensitized patients with calculated panel reactive antibodies greater than 80 exhibit antibody binding to Class I swine leukocyte antigen (SLA-1). This cross-reactivity is most prevalent in patients with HLA-A sensitivity (74) and appears to target a common cross-reactive group involving a conserved lysine residue found in every listed SLA-1 protein. Serum from sensitized patients with Class II anti-HLA DQ4, DQ5, and DQ6 antibodies also show reactivity to some porcine class II SLA DQ alleles (75). The reactivity to SLA DQ may also be localized to a defined epitope. These results suggest that, as in allotransplantation, recipient preformed and induced antibodies to swine MHC antigens may contribute to AMR after clinical CXtx.

**Figure 1:**
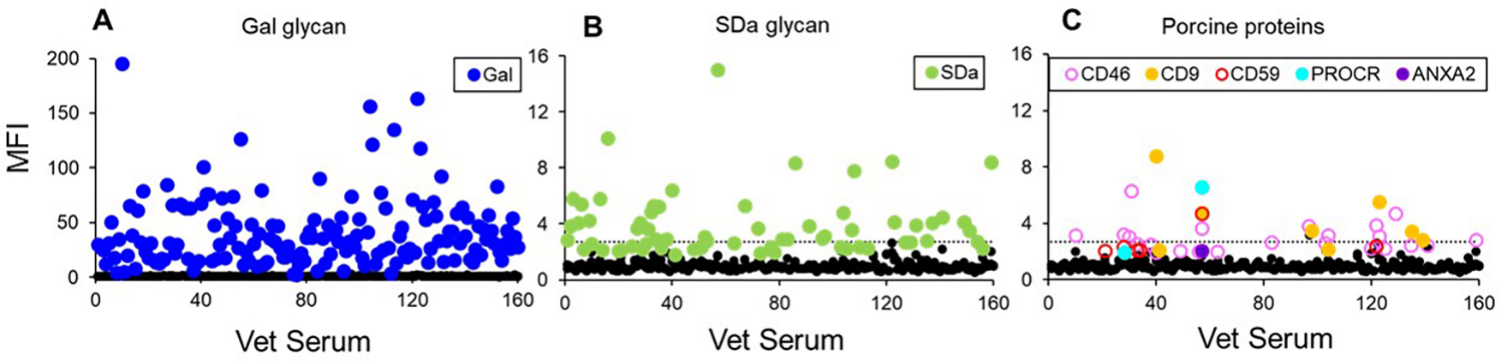
**(A)** Summary of swine veterinarian serum IgM binding to glycan **(A,B)** and protein antigens **(C)**. **(A)** Anti-Gal IgM reactivity for 160 swine veterinarian serum samples. All samples had anti-Gal reactivity. **(B)** IgM binding to SDa with 65 serum samples showing antibody binding greater than three standard deviations above background. **(C)** IgM binding to protein antigens with 28 swine veterinarian serum samples having antibody binding greater than three standard deviations above background. The dotted line in **(B,C)** represents five standard deviations above the average MFI of all HEK control cells. Adopted from (77).

In allotransplantation, polymorphic donor MHC antigens including HLA Class I and Class II proteins were identified as the predominant targets of pre-existing and induced anti-donor antibodies. In Xtx, donor and recipient polymorphism is not unique to porcine MHC antigens but is expanded to include nearly all EC proteins (76). This greatly expands the landscape of non-Gal protein antigens that can contribute to AMR after clinical CXtx. We previously identified the porcine proteins CD9, CD46, CD59, protein C receptor (PROCR), and annexin A2 (ANXA2) as immunogenic in NHPs after CXtx (60). Recently, we demonstrated human antibody binding to these proteins in serum from veterinarians (*n* = 160) specializing in swine medicine (77). We found that all samples contained high levels of anti-Gal IgM with approximately 40% of the samples showing both anti-Gal and anti-SDa IgM reactivity (Figures 1A,B). In addition, approximately 10% of the veterinarian serum samples exhibited IgM binding to one or more of the pig proteins (CD9, CD46, CD59, PROCR, and ANXA2) (Figure 1C). These results emphasize the broad array of protein antigens, including swine SLA, which may be immunogenic and contribute to AMR of clinical Xtx without highly effective immune suppression or some degree of tolerance.

### Further donor genetic modification

Even with the establishment of triple knockout pigs with reduced antigenicity, further donor genetic modification has been applied to address apparent biochemical incompatibilities and augment organ resistance to AMR. In addition to the human complement regulators (CD46, CD55, and CD59), discussed previously, human transgenes expressing human thrombomodulin (TBM) and endothelial cell protein C receptor (EPCR) have been introduced to correct thromboregulatory deficiencies (see below, Immune suppression with co-stimulation blockade). Additionally, human CD47 has been added to the pig genome to suppress the activation of phagocytic macrophages and the infiltration of T cells. Human heme oxygenase-1 has been added to generally suppress oxidative injury, and in some instances, the porcine growth hormone receptor (GHR) has been disrupted to reduce the rate of donor growth and limit donor organ size. In general, the benefits of these additional human transgenes have not been as systematically demonstrated as was done for human complement regulators (25, 45, 78). Incorporation of human TBM into the GTKO; CD46 donor genetic has been described to improve heterotopic CXtx survival (24, 28), and this donor genetic has been frequently used for preclinical orthotopic CXtx (39–43). The inclusion of a large number of genetic modifications has not prevented AMR, and further study would be prudent to gauge the value of these transgenes (79).

### Immune suppression with co-stimulation blockade

Immune suppression progressed (Table 2) from the early protocols, relying in part on the ablative effects of cyclophosphamide, to protocols using clinically approved reagents and highly effective co-stimulation blocking protocols. With each improvement in immunosuppression, median preclinical heterotopic CXtx graft survival increased from 33 days with cyclophosphamide (30) to 96 days with tacrolimus and sirolimus (36) and 298 days using an anti-CD40 co-stimulation blocker (9). Co-stimulation blockade is now currently the immune strategy of choice because of its effectiveness in suppressing an induced antibody response and achieving longer-term graft survival.

Early co-stimulation blocking immune suppression relied on using an anti-CD154 antibody, which was known to promote thrombocytopenia in allotransplantation due to platelet CD154 expression and Fc-receptor activation (80, 81). Alone and especially when coupled to cobra venom factor, the use of anti-CD154 was associated with systemic inflammation and consumptive coagulopathy after CXtx (37, 46). In light of *in vitro* studies of pig ECs, these studies emphasized the potential impact of thromboregulatory incompatibilities which might render the pig organ inherently thrombogenic (82) and thereby supported expressing human TBM, EPCR, and CD47 in the donor organ to address molecular incompatibilities. Whether all these genes are necessary requires further research, but what is clear is that on the first reported instance of using an alternative anti-CD40 (2C10R4), co-stimulation blocker evidence of thrombocytopenia and consumptive coagulopathy after CXtx of GTKO donor pigs expressing only human CD46 and TBM was minimized (10). Using an immune suppression strategy based on T- and B-cell depletion with maintenance therapy consisting of high-dose anti-CD40 and MMF heterotopic CXtx survival increased to a maximum of 945 days (median 298 days) (9). Immune suppression with co-stimulation blockade using this antibody or alternatively PASylated Fc fragments of anti-CD154 has achieved a median survival of heterotopic and orthotopic GTKO donors expressing human CD46 and TBM which commonly meets or exceeds 3 months (83–85).

B-cell induction and the development of AMR are a complex process with many points of potential intervention that have not yet been explored in Xtx (86). Even under the best co-stimulation blocking immune suppression regimens, long-surviving xenografts can reject or show evidence of antibody deposition and ongoing immune injury (87–89). This suggests that current immune suppression is not fully effective and that the addition of other agents may be necessary for achieving routine and durable survival beyond 1 year. There are a variety of Bruton's tyrosine kinase inhibitors, which affect signal transduction from the B-cell receptor, IL-6 antagonists essential for differentiation of B cells, B-cell-specific cytokine antagonists for B-cell activating factor (BAFF), and a proliferation-inducing ligand (APRIL) and even CAR-T therapy targeting memory B cells which might be used to augment co-stimulation blockade. Many of these drugs are already approved for other therapies, but it is up to the Xtx research community to explore their utility in the context of preclinical Xtx studies.

## Preclinical cardiac xenotransplantation in NHPs: orthotopic transplantation

Once a median of 96-day heterotopic CXtx survival was achieved, attention turned to reproducing this level of efficacy in a life-supporting orthotopic transplant model. Early orthotopic transplants using wild-type pig hearts into NHPs were not effective (90, 91). Without any treatment graft, survival was <24 h (Table 3). When pretransplant organ perfusion was used to deplete circulating antibodies, median survival was limited to 5.5 days. When antibody absorption was coupled with extensive irradiation and immune suppression, median survival was only extended to 18 days (91). More recent orthotopic transplant studies using GE donor organs expressing human complement regulatory gene CD55 (hDAF) also met with highly variable and limited success (92–97). Using a human CD46 transgenic donor with the inclusion of Gal-polymers to block anti-Gal antibodies, our group achieved maximum orthotopic survival of 57 days (39) which remained the longest-surviving orthotopic cardiac xenotransplant until 2018 (Table 3).

### Perioperative cardiac xenograft dysfunction

These early orthotopic studies clearly demonstrated the ability of the pig heart to sustain the circulation in NHPs, but they also unmasked an unanticipated impediment to clinical CXtx, not apparent from heterotopic studies. Each research group reported variable perioperative mortality ranging from 40% to 60% within the first 48 h of orthotopic CXtx. Xenograft failure in this time period was not due to HAR and showed a gene profile that was distinct from graft rejection but suggestive of ischemia–reperfusion injury (98). At explant, cardiac xenotransplants showed vascular antibody deposition but otherwise normal myocardial histology (14, 99, 100). This early graft failure, which we termed perioperative cardiac xenograft dysfunction (PCXD), was attributed to primary organ dysfunction from ischemia–reperfusion injury but was recoverable to normal heart function in a small number of cases (39, 98). PCXD does not appear to be moderated by different donor genetics or pretransplant immune suppression (99). At this high frequency, PCXD represented a significant barrier to clinical CXtx.

### Prolonged orthotopic cardiac xenograft survival

In 2018, the Munich group addressed the issue of PCXD in a landmark study by using a non-ischemic continuous organ perfusion (NICP) method and cardioplegia developed by Steen and colleagues (85, 101). This new organ preservation method coupled with GTKO donors expressing human CD46 and TBM resulted in superior and more predictable outcomes with 3-month median survival (Table 3). This study was the first to achieve orthotopic CXtx survival consistent with ISHLT 2000 criteria for clinical efficacy (84, 85). PCXD was consistently avoided and graft rejection was prevented using an anti-CD40 and pasylated anti-CD154 Fab fragment co-stimulation blockade supplemented with temsirolimus and antihypertensive drugs. Subsequently, studies in other centers confirmed the prevention of PCXD using non-ischemic *ex vivo* perfusion and achieved survival up to 9 months in NHPs using GE donor pigs with further variable gene modifications (83, 99, 102).

Across the arch of heterotopic and orthotopic preclinical CXtx research (Tables 2, 3), the recent success of orthotopic CXtx appears to be due to two main developments. The first was the progressive improvement in immune suppression, principally the adoption of co-stimulation blockade developed at NIH and subsequently at the University of Maryland (UMD) (9, 10, 103). The second was the development of NICP organ preservation pioneered by the Munich group (85, 89, 100, 101). Less evident but contributing to success are the years of experience these groups have accumulated in developing methods for postoperative monitoring and management of NHP recipients. This suggests that effective donor preservation, immune suppression, and careful postoperative monitoring and management are important contributors to prolonged xenograft survival. These same components, learned from preclinical CXtx, are applied for successful clinical CXtx.

In NHP recipients, the most consistent CXtx results come from the Munich group using GTKO; CD46; hTM donor pigs (Table 3). These donors are not a perfect cross-match as NHPs express antibodies to SDa and may also express antibodies to porcine SLA. Despite imperfect matching, the Munich group has shown consistent recipient survival beyond 3 months. Isolated longer survival in NHPs using donors with multigene modifications has been reported by the UMD group (83). The inclusion of further donor genetic modifications to eliminate Neu5Gc and SDa glycans minimizing human antibody reactivity is likely to help improve long-term clinical organ survival. Additional genetic engineering to include the expression of human transgenes has been used in preclinical and clinical transplants, but these genes do not appear sufficient to prevent rejection; rather, their benefits, if any, are dependent on effective immune suppression (99). The practical utility of these other GE modifications is unproven and merits further study.

## Clinical cardiac xenotransplantation

Based on the substantial improvement of survival in preclinical CXtx, UMD has performed two clinical CXtxs under FDA single-patient expanded access protocols (EAPs). In each case, a donor organ with all three glycan deletions (Gal, SDa, and Neu5Gc), a pig growth hormone receptor deletion (GHRKO), and a series of human transgenes (hCD46, hCD55, hTBM, hEPCR, hCD47, hHO-1) were used (a “10-gene pig”).

The first patient was a 57-year-old male with refractory heart failure who was not a candidate for allotransplantation or durable mechanical circulatory support (7, 104). He was transplanted with a 10-gene GE donor pig heart preserved with an XVIVO heart perfusion system and treated with an immune suppressive regimen based on previous preclinical studies. Immune suppression included induction corticosteroid therapy, B-cell depletion with rituximab, systemic complement inhibition with a C1 esterase inhibitor, and co-stimulation blockade with an investigational anti-CD40 antibody (KLP-404, Kiniksa Pharmaceuticals). Maintenance therapy consisted of tapering corticosteroids, ATG for the first 3 days, daily mycophenolic mofetil (MMF), and repeated anti-CD40 to maintain a targeted serum concentration. The patient was weaned from extracorporeal membranous oxygenation (ECMO) by postoperative day (POD) 4, and the cardiac xenograft showed good function with an LVEF of 55%. On POD 34, an endomyocardial biopsy (EMB) showed mild interstitial edema with some mild C3d, C4d, IgG, and IgM deposition without apparent EC damage. Serum anti-pig antibody levels remained low until POD 47 after IVIG administration. The patient had abrupt diastolic heart failure on POD 49 requiring ECMO. An EMB taken that day showed interstitial edema, disorganized endothelium, and C4d, IgG, and IgM deposition with extravasation of erythrocytes. The patient was treated with plasma exchange for AMR and given a second dose of IVIG. An additional EMB on POD 56 showed AMR of ISHLT Grade 1 with 40% myocyte necrosis. The patient was not able to be weaned from ECMO, and life support measures were discontinued on POD 60. This patient's clinical results were complicated by the degree of heart failure and his clinical state prior to transplant, the use of IVIG which contained anti-pig antibody (105), and the emergence of porcine CMV infection of the donor heart after transplant. Postoperative porcine CMV infection has been shown to limit xenograft survival in NHPs (84).

The second patient was a 58-year-old male with progressive heart failure due to ischemic cardiomyopathy (8). The patient was declined for allotransplant by two centers due in part to severe peripheral and central atherosclerotic vascular disease and a recent gastrointestinal bleed. As with the first patient, the donor heart was from a 10-gene pig subjected this time to more extensive serology and molecular testing to ensure a porcine CMV negative status. The donor heart was preserved using the same XVIVO perfusion system, and the xenograft functioned well immediately after reperfusion. Immune suppression was largely the same as the first patient except a different investigational anti-CD40 antibody was used (Tegoprubart, Eledon Pharmaceuticals). On POD 4, the patient was reoperated to address a mediastinal hemorrhage from a pacing lead. This required significant administration of blood products including fresh-frozen plasma (FFP) and packed red blood cells. On POD 7, the patient was reintubated after a brief respiratory cardiac arrest. An EMB on POD 13 **s**howed prominent vascular staining for IgG, IgM, C3d, and C4d with evidence of EC activation consistent with AMR. Although serum anti-pig antibody was not elevated at this time, therapeutic plasma exchange was performed on POD 14 using a 50% albumin and 50% FFP replacement. Multiple batches of FFP were subsequently shown to contain anti-pig antibodies. On POD 29, the patient had acute hemodynamic decompensation requiring resuscitation and increased vasopressor support. An EMB on POD 30 showed increased vascular IgM and IgG and complement deposition with diffuse EC activation. The patient's condition continued to deteriorate, and he was placed on ECMO on POD 31. On POD 35, a series of plasma exchanges were initiated to treat apparent AMR using 100% albumin replacement. By POD 40, there was no improvement in cardiac function, and the patient could not be weaned from ECMO. The patient opted for comfort care at that time.

The results of these groundbreaking clinical CXtxs highlight several remaining critical concerns facing Xtx if consistent and enduring organ survival is to be achieved. In both patients, treatment for AMR, namely, plasma exchange to deplete circulating antibody, included the infusion of blood products: IVIG or FFP. These products are used as sources of IgG to block Fc-receptor function and also as replacement proteins to limit infectious risk, hemodynamic instability, and bleeding. This treatment is used for AMR in allotransplant recipients. In retrospect, it became clear that both IVIG and FFP contained anti-pig antibodies which likely contributed to cardiac xenograft injury in these severely ill patients. This highlights the essential need for effective antirejection therapy to reverse AMR. Also, in each of these cases, the patient's immune response was monitored by multiple parameters including phenotyping for B- and T-cell subsets, EMB for histology and immunohistology, and assessing serum anti-pig antibody levels by flow cytometry staining porcine aortic ECs or peripheral blood mononuclear cells of the donor or a clonally matched 10-gene pig. Complement-dependent cytotoxicity was similarly determined using donor porcine aortic ECs. These cell-based cross-match assays (CDC and flow cytometry) do not have the sensitivity of solid phase immunoassays (Luminex) and as such may not detect anti-pig antibodies, including anti-SLA antibodies, in circulation or in therapeutic blood products. Cardiac function and injury were monitored by echocardiography, serum troponin, and weekly blood samples for xenograft-derived cell-free DNA. Whether these analyses were performed in real time, near real time, or in retrospect is not clear, but the results indicate that none of the assays was sufficiently predictive of AMR to allow timely intervention. AMR was first evident as vascular antibody and complement deposition in EMBs on POD 34 (first patient) and POD 13 (second patient). At that time in both patients, there was scant corroborating evidence for AMR in the form of elevated serum antibody or cytotoxicity, changes in B- or T-cell profiles, or increased evidence of cardiac damage measured by troponin or cell-free DNA. Since the bulk of anti-pig antibodies in these patients appear to be bound to the graft, new methods to diagnose rejection after CXtx will require a major diagnostic development to identify circulating non-Gal antigens and establish highly sensitive assays to detect these antibodies. Immunological analysis of IVIG and FFP may provide new insights into specific non-Gal antigens that contribute to rejection after clinical CXtx.

## Human decedent cardiac xenotransplantation model

In June and July 2022, a team at New York University Langone performed two CXtx into human decedent recipients (106). Decedent recipients for Xtx are humans who have been declared brain dead but are not suitable to be organ donors. They are ventilator-dependent and have a beating heart and stable hemodynamics. Their families give permission for whole body donation for Xtx research. With an increase in donation after brain death (DBD), there has been an increase in clinical experience in managing decedents for several days as organ allocation and recovery are performed (107).

The two NYU decedent CXtx recipients received donor pig hearts with 10 GE edits (GTKO, CMAHKO, B4TKO, GHRKO, CD46, CD55, hTBM, CD47, EPCR, HO-1). These studies were designed to test the acute (66 h) hemodynamic function of the pig heart and to determine if these 10-gene donor hearts were resistant to hyperacute rejection (108). In the first case, the donor heart was undersized and required increased inotrope and vasopressor support. The left ventricular ejection fraction (LVEF) of this heart decreased from 70% to 40%–45% by postoperative day (POD) 3. In the second case, the donor heart was more appropriately sized and showed stable cardiac function (LVEF 75% on POD3) without increased inotropic support. At explant, both hearts showed normal myocardium but also areas of subendocardial hemorrhage and patchy myocyte coagulative necrosis. Patchy inflammation was associated predominantly with macrophages and few eosinophils. The first heart showed areas of coagulative necrosis associated with myocyte calcification scattered throughout the myocardium. The first heart also showed sparse CD3+ lymphocytic infiltration not present in the second heart. Mild deposition of C4d-positive myocytes was evident on POD 2 and was more pronounced at explant (POD 3) in the first heart. In the second heart, C4d deposition was faint or rare throughout the transplant. The deposition of C4d was not indicative of AMR but rather may have reflected the consequence of ischemic injury after transplant. In both cases, cardiac preservation used traditional cardioplegic perfusion and cold storage prior to transplant and did not use NICP. This suggests, especially for the first decedent, that both hearts may have been subject to some degree of PCXD.

Extensive serial multi-omic profiling of these decedent CXtx recipients was performed to define, in molecular terms, the early human responses after CXtx (109). This analysis included single-cell transcriptomics, lipidomics, proteomics, and metabolomic profiling of decedent blood samples collected at 6 h intervals. Histologic and transcriptomic analysis was also performed on the cardiac tissue. These analyses showed a clearly distinct response between the first and second decedents. The first decedent showed evidence of a pronounced immune response associated with a rise in T cells and NK cells, a spike in B-cell induction, and changes in metabolism and gene expression consistent with ischemic injury and PCXD. These changes were minimally present in the second decedent.

The decedent model offers a unique opportunity to define authentic human responses to CXtx which cannot be obtained from studies in NHPs. The initial CXtx decedent studies were of limited duration, but a pig renal Xtx into a decedent has been reported for circa 60 days (106). If future decedent CXtx recipients can be maintained for this duration, then the decedent model could provide critical insights into authentic human immune responses to pig hearts for a meaningful period. As well as optimizing immune suppressive drug doses and strategies, this duration of study could make possible identification of new non-Gal antigens causing AMR and allow for its diagnosis. Importantly the decedent model provides an opportunity to refine postoperative clinical management, test xeno-specific procedures for treating AMR, and use state-of-the-art unbiased molecular analysis that is optimized for human samples to characterize in detail the human response to CXtx.

The decedent model, requiring a dedicated hospital clinical-level intensive care unit, is estimated to be far more expensive than preclinical NHP studies which will limit its use to only the largest transplant centers. Decedent studies may also be affected by pathophysiological changes associated with brain death (110). These changes are generally transitory, and research on brain death donors has developed methods of modulating hemodynamic instability (107). To avoid a Shelley-esque image (111), decedent studies must be carefully arranged, managed, maintained, and supported with full transparency and compliance with the Uniform Determination of Death Act and institutional oversight. Even with studies lasting up to 3 months, the decedent model is limited to short-duration immune responses. Studies showing prolonged effective immune suppression in NHPs will still be helpful in demonstrating long-term graft durability.

## Cardiac xenotransplantation: remaining challenges

Preclinical studies have optimized CXtx for NHPs with current orthotopic survival of up to 9 months. This result has required a Herculean effort over the last 30 years in animal care, genetic engineering, and extensive testing of experimental drug regimens. The main challenge now is to translate this experience with NHPs into a successful clinical CXtx program. To accomplish this, it is clear that further clinical optimization will be needed in immune suppression, the diagnosis and treatment of AMR, pathogen-free donor pig production and testing, and a better understanding of the significance of pig organ overgrowth on long-term CXtx survival (Table 4). The decedent model may be preferred for certain studies as decedents more closely approximate the immune and pharmacokinetic drug responses that will be encountered in clinical Xtx. Other challenges for CXtx, such as studies optimizing long-term survival, will require ongoing research in NHPs. Xenotransplantation is at a crossroads. The transition from preclinical to clinical CXtx has not progressed as smoothly as the preclinical studies might have suggested but we believe that careful deliberative studies in both NHPs and decedent humans will be complementary to overcome the remaining challenges before full clinical studies are warranted.

**Table 4:** Challenges for clinical cardiac xenotransplantation.

Challenge	Key problems
Immune suppression	• Regulatory approval for key components (co-stimulation blocker) • Defining an optimal combination of drugs to control AMR. • Defining the optimal drug dosing to control AMR • Optimizing long-term survival
Diagnosis and treatment of AMR	• Identifying diagnostic non-Gal antigens • Establishing new highly sensitive assays for non-Gal antibody detection • Establishing effective xeno-specific methods to treat AMR
Specific pathogen-free donor pigs	• Stringent high health donor pig standards must be maintained • Testing for porcine CMV must include both molecular and serology CMV testing at multiple time points prior to transplant
Organ growth or rejection	• Determining the degree of intrinsic organ growth contributes to post-transplant cardiac xenograft growth
